# Polymorphic Ventricular Tachycardia Associated with High-Dose Methadone Use

**DOI:** 10.1155/2020/4504657

**Published:** 2020-09-23

**Authors:** Weng-Chio Tam, U-Po Lam, Toi-Meng Mok, Tou Chang, Wa Ho, Mario Alberto De Brito Lima Evora

**Affiliations:** Department of Cardiology, Centro Hospitalar Conde São Januário, Macau, SAR, China

## Abstract

Methadone is a well-tolerated drug that has been used for pain control and the treatment of opioid addiction. However, some fatal cardiac side effects have been reported previously, including ventricular arrhythmia, stress cardiomyopathy, and coronary artery disease. We reported a middle-aged woman receiving high-dose methadone whom was presented with QT prolongation and torsade de pointes. We replaced the methadone with benzodiazepine and gave lidocaine use simultaneously. Thus, QT interval was shortened within the normal limit. Methadone-induced torsade de pointes is a rare but serious event, and QT interval should be monitored periodically to prevent this fatal adverse event, especially some patients with high-dose methadone use.

## 1. Introduction

Long QT syndromes can be categorized as either congenital or acquired etiologies. QT prolongation is manifested by the delayed cardiac repolarization phase resulting from transmembrane potassium, sodium, and calcium ion current alteration [1]. These may cause fatal ventricular arrhythmia and sudden cardiac death. Acquired long QT syndrome is known to be associated with some antibiotics, antihistamines, electrolyte imbalance, and metabolic disorders. Methadone is a synthetic opiate most commonly used for the pain control and treatment of heroin addiction. It may also block the cardiac potassium ion current and induce QT prolongation and dispersion, especially high-dose methadone [2]. The prevalence of methadone-induced QT prolongation and torsade de pointes may also be underestimated clinically. Therefore, we investigate the association of high-dose methadone and fatal ventricular arrhythmia in this report.

## 2. Case Presentation

**Figure 1:**
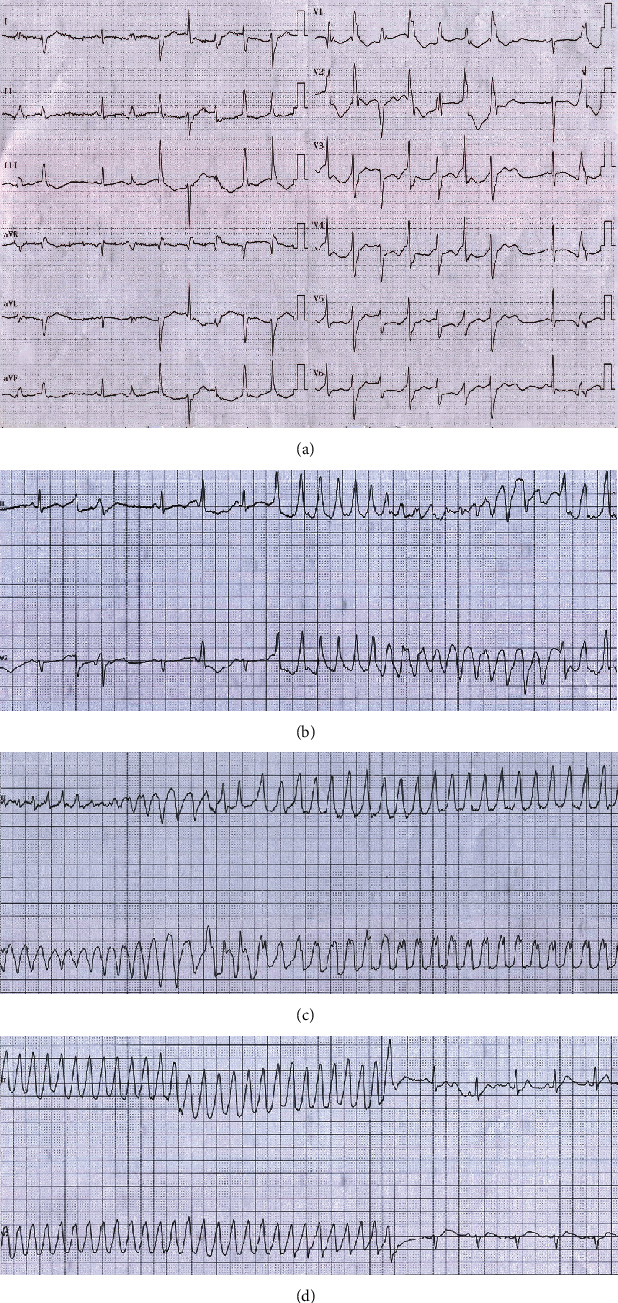
(a) 12-lead ECG revealed sinus rhythm with frequent PVCs. (b) Telemetry showed polymorphic premature ventricular contractions and R on T, and TdP was initiated. (c) QRS amplitude varied, and QRS complexes appeared to twist around the baseline, suggestive of TdP. (d) Self-termination of TdP.

**Figure 2:**
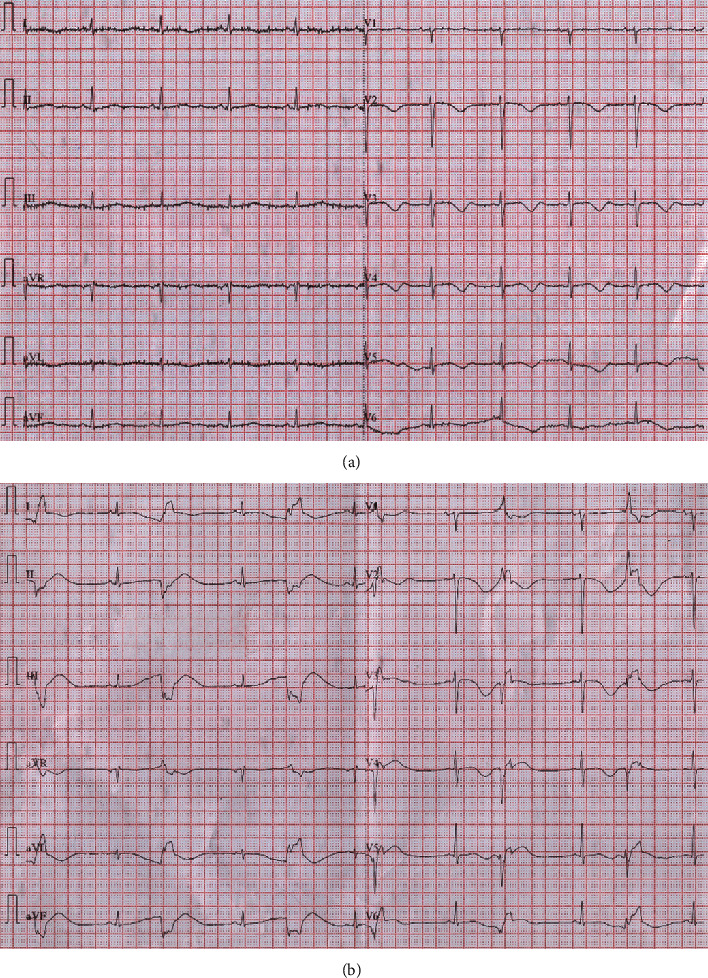
(a) After termination of TdP, repeated ECG revealed sinus rhythm with significant QT prolongation and QT interval was about 600 milliseconds. (b) ECG showed sinus rhythm with bigeminy PVC and R on T.

**Figure 3:**
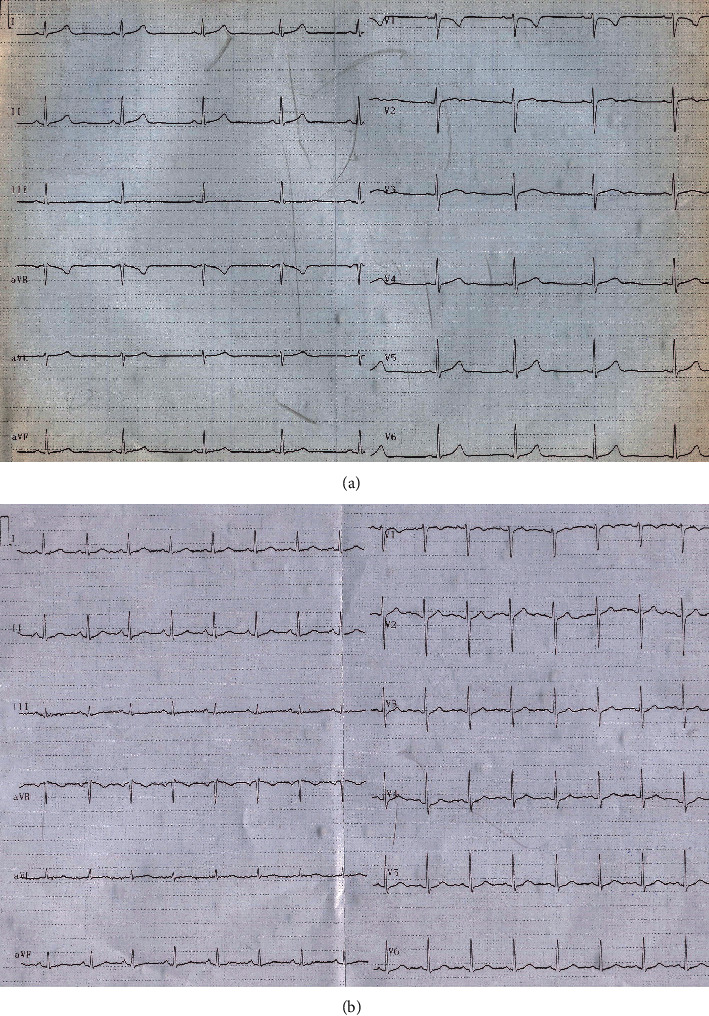
(a) The ECG without methadone use showed normal QT interval. (b) After discontinuation of methadone use, QT interval was shortened to near 460 milliseconds.

A 44-year-old female patient presented herself with sudden onset of impaired consciousness at our emergency room. She had an underlying disease of microcytic anemia. She was also a methadone user for opioid addiction, receiving 130 mg/day. She initiated methadone use about 6 months ago. Otherwise, she denied any other medication use. On arrival, physical examination showed body weight: 55 kg, body temperature: 36.5°C, blood pressure: 114/70 mmHg, and pulse: 124/min. 12-lead electrocardiography (ECG) revealed sinus rhythm with frequent premature ventricular contractions (PVCs) (Figure 1(a)). The result of chest X-ray showed cardiomegaly with pulmonary edema. Echocardiography revealed preserved left ventricle systolic function with mild tricuspid regurgitation and grade III diastolic dysfunction. The level of potassium, calcium, and magnesium were within normal limit (potassium: 3.7 mmol/L (3.4-4.5), calcium: 2.16 mmol/L (2.15-2.5), and magnesium: 0.8 mmol/L (0.66-0.99)).Recurrent conscious disturbance was found about several minutes after the first episode. ECG monitor showed polymorphic PVCs, R on T, and QT prolongation, suggestive of torsade de pointes (TdP) with unstable hemodynamic status (Figures 1(b) and 1(c)). After three times of 200 J biphasic cardioversion defibrillation, ECG rhythm was converted to sinus rhythm spontaneously by herself (Figure 1(d)). Intravenous magnesium sulfate was used. Repeated ECG showed sinus rhythm with obvious QT prolongation (QT interval > 600 milliseconds) (Figure 2(a)) and intermittent bigeminy PVC with R on T (Figure 2(b)). We gave her lidocaine to treat the potentially fatal ventricular arrhythmia because of its class IB antiarrhythmic effect. We gave magnesium and potassium supplements to prevent hypokalemia and hypomagnesemia, simultaneously to suppress early afterdepolarizations. We reviewed her medical and family history. She denied any history of syncope or premature family history of sudden death. Moreover, the previous ECG without methadone use was documented about 2 years ago (Figure 3(a)). It showed sinus rhythm with normal QT interval. According to the QT interval of documented ECG and medical background of high-dose methadone use, acquired long QT syndrome was suspected eventually. We discontinued the methadone and replaced it with benzodiazepine. Acute coronary syndrome was excluded because coronary computed tomography showed insignificant coronary artery disease with eccentric plaque over the left anterior descending artery. No recurrence of ventricular arrhythmia was noted after discontinuation of methadone use. QT interval was shortened to near 460 milliseconds within 2 weeks (Figure 3(b)).

## 3. Discussion

Methadone is a synthetic opiate which has been commonly used for the pain management. It is a mu-receptor and may act as antagonist at the NMDA receptor. Physicians also use this medication either for opiate detoxification or maintenance to help people with opioid dependence. However, there are similar side effects to those of opioids, commonly including dizziness, sleepiness, vomiting, and sweating. More serious risks include respiratory depression and fatal cardiac arrhythmia. The incidence of adverse effect increases with higher doses [3], and high dose was defined as >100 mg daily methadone use [4]. Unexpected death could be associated with methadone which had lead to clinical safety concern by the U.S. Food and Drug Administration in 2006 [5]. Furthermore, recent studies indicated that methadone may be associated with polymorphic ventricular tachycardia and QT prolongation-induced TdP [3, 6]. The incidence of methadone-associated TdP was about 0.06/100 patient-years [7]. Patients with QT prolongation while using methadone should be cautioned and focused by clinicians as this could be one necessary precondition for the result of sudden cardiac death.

Methadone inhibits the human ether-a-go-go-related gene (hERG1) encoding for a potassium channel protein that regulates Ikr. This Ikr blockage may cause prolonged duration of the phase III of the ventricular action potential. It finally delays repolarization and represents as QT interval prolongation [8]. Moreover, it can trigger early afterdepolarizations due to the activation of L-type calcium channels and sodium-calcium exchange channel. According to the triggered activity from the early afterdepolarization, ectopic beats and premature ventricular extrasystoles can be generated to initiate TdP [9]. In addition to its effect of channelopathy, methadone use may be associated with the development of bradycardia because of its anticholinesterase properties. Increased QT dispersion and negative chronotropy has also been proven to be possible mechanism of methadone-induced TdP [10]. Moreover, other risk factors include hypokalemia, hypomagnesemia, hypocalcemia, and female. It is worth noting that concomitant use of psychiatric medication or anti-HIV medication can affect the drug-drug interaction and predispose the effect of QT prolongation from methadone [9].

The treatment of methadone cardiotoxicity depends on the patient's symptoms and signs. For asymptomatic patients, correction of electrolytes imbalance should be considered. Dose reduction or substitution of methadone is also reasonable. Some alternatives of methadone may also be considered including buprenorphine, naltrexone, or slow releasing morphine [11]. For patients with ventricular tachyarrhythmia, in addition to cessation of offending agents and electrolyte correction, magnesium sulfate is the first-line therapy. Temporary pacing or intravenous isoproterenol may be considered to prevent bradycardia. Intravenous lidocaine or oral mexiletine is also an alternative medication for some patients with recurrence of tachyarrhythmia [12].

In conclusion, although methadone is commonly used for the treatment of pain and opioid addiction, its cardiotoxicity, especially fatal ventricular arrhythmia, should be treated with caution. For some patients with hypokalemia or combination of medication that prolong the QT interval, physicians should monitor the QT interval before and during the methadone use, especially when high-dose methadone is prescribed.
